# The association of multiple anti-hypertensive medication classes with Alzheimer’s disease incidence across sex, race, and ethnicity

**DOI:** 10.1371/journal.pone.0206705

**Published:** 2018-11-01

**Authors:** Douglas Barthold, Geoffrey Joyce, Whitney Wharton, Patrick Kehoe, Julie Zissimopoulos

**Affiliations:** 1 Comparative Health Outcomes, Policy, and Economics (CHOICE) Institute, Department of Pharmacy, University of Washington, Seattle, Washington, United States of America; 2 School of Pharmacy, Schaeffer Center for Health Policy and Economics, University of Southern California, Los Angeles, California, United States of America; 3 Department of Neurology, Emory University School of Medicine, Atlanta, Georgia, United States of America; 4 Bristol Medical School, Translational Health Sciences, University of Bristol, Bristol, United Kingdom; 5 Price School of Public Policy, Schaeffer Center for Health Policy and Economics, University of Southern California, Los Angeles, California, United States of America; International University of Health and Welfare, School of Medicine, JAPAN

## Abstract

**Background:**

Antihypertensive treatments have been shown to reduce the risk of Alzheimer’s disease (AD). The renin-angiotensin system (RAS) has been implicated in AD, and thus RAS-acting AHTs (angiotensin converting enzyme inhibitors (ACEIs), and angiotensin-II receptor blockers (ARBs)) may offer differential and additional protective benefits against AD compared with other AHTs, in addition to hypertension management.

**Methods:**

In a retrospective cohort design, we examined the medical and pharmacy claims of a 20% sample of Medicare beneficiaries from 2007 to 2013, and compared rates of AD diagnosis for 1,343,334 users of six different AHT drug treatments, 65 years of age or older (4,215,338 person-years). We compared AD risk between RAS and non-RAS AHT drug users, and between ACEI users and ARB users, by sex and race/ethnicity. Models adjusted for age, socioeconomic status, underlying health, and comorbidities.

**Findings:**

RAS-acting AHTs were slightly more protective against onset of AD than non-RAS-acting AHTs for males, (male OR = 0.931 (CI: 0.895–0.969)), but not so for females (female OR = 0.985 (CI: 0.963–1.007)). Relative to other AHTs, ARBs were superior to ACEIs for both men (male ARB OR = 0.834 (CI: 0.788–0.884); male ACEI OR = 0.978 (CI: 0.939–1.019)) and women (female ARB OR = 0.941 (CI: 0.913–0.969); female ACEI OR = 1.022 (CI: 0.997–1.048)), but only in white men and white and black women. No association was shown for Hispanic men and women.

**Conclusion:**

Hypertension management treatments that include RAS-acting ARBs may, in addition to lowering blood pressure, reduce AD risk, particularly for white and black women and white men. Additional studies and clinical trials that include men and women from different racial and ethnic groups are needed to confirm these findings. Understanding the potentially beneficial effects of certain RAS-acting AHTs in high-risk populations is of great importance.

## Introduction

An estimated 5.5 million Americans have Alzheimer’s disease (AD), which is the most common cause of dementia [1]. The lack of disease-modifying treatments for AD has focused attention to commonly prescribed medications for other conditions (i.e., so-called drug repurposing or repositioning) that may reduce risk and delay onset of AD, benefitting individuals, families, and societies [2]. Vascular disease, and in particular hypertension, has long been recognized as a risk factor for AD and other dementias [3]. Hypertension is highly prevalent among United States adults (29%), is higher for African Americans than whites (44% compared to 32%), and rises with age for men (64% ages 65–74, and 66.7% ages 75 and older) and for women (69.3% ages 65–74, and 78.5% ages 75 and older) [4, 5].

Antihypertensive (AHT) medications are commonly prescribed for adults with chronic high blood pressure, spanning six different widely used therapeutic classes: two renin-angiotensin system (RAS) acting classes (angiotensin converting enzyme inhibitors (ACEIs) and angiotensin-II receptor blockers (ARBs)), and four classes that work primarily via non-RAS mechanisms (beta blockers (BBLs), calcium channel blockers (CCBs), loop diuretics (LDs), and thiazide-like diuretics (TDs)). In the US in 2014, among the civilian, non-institutionalized population age 65 years and over, 24.1% were prescribed ACEIs, 12.2% ARBs, 30.5% BBLs, 17.8% CCBs, and 21.1% diuretics within a single month [6].

There is observational evidence showing that AHT treatments, regardless of drug class, are protective against cognitive decline and incident dementia [3]. Meta-analyses reviewing evidence from both observational studies and randomized controlled trials (RCTs) have shown AHT drug treatment may be protective against incident dementia or cognitive decline but results were inconsistent due to variation across study designs, follow-up periods, patient populations, the definition of hypertension status, and outcomes where, in the majority of cases, cognitive function was a secondary outcome or part of a substudy [3, 7–11]. Some observational studies suggest that RAS-acting AHTs are more protective against AD than other AHTs [12]. Human and animal studies suggest that RAS medications may modulate numerous protective mechanisms against AD (see Kehoe, 2018 for review), independent of blood pressure effects [13–18]. In a sample of veterans, predominately white males, Li et al (2010) provided evidence that within RAS-acting AHTS, ARBs were more protective against dementia than one ACEI (lisinopril) [19]. Other studies, including the randomized controlled trials ONTARGET, TRANSCEND, and PROFESS, have found no effect of ACEIs and ARBs on cognitive function, but no direct comparisons were made between these RAS-acting classes and non-RAS acting classes [20, 21]. Several more recent studies showed that individuals taking RAS-acting antihypertensives exhibited slower disease progression, likely via less AD related neuropathology, particularly neurofibrillary tangle pathology, than individuals taking non-RAS-acting antihypertensives [12, 22–25]. It is hypothesized that ARBs are more protective than ACEIs because of ACEI’s role in amyloid-β degradation (shown in in vitro and in animal studies, see Kehoe 2018 for review), but existing RCTs, where AD was the targeted outcome, are not yet completed [26–28]. Studies of AHT drugs in people with cardiovascular morbidities have not reported cross-class differences in relation to secondary investigations on cognition and dementia [3, 7, 8, 12, 19, 29, 30].

Despite important advances in the potential RAS-AD link, and efforts to enroll minorities in clinical trials, there have been limited population-based investigations on these relationships in samples with substantial representation of women and racial/ethnic minorities, providing little evidence on cross-class differences for specific subpopulations that could inform the generalizability of various treatments on AD risk. Investigations of large datasets can help close this research gap. It is well documented that RAS-acting agents function differently in African Americans compared to Caucasians, with higher endogenous sodium and lower renin levels for African Americans. Because African Americans are at higher risk for AD, and it is unknown whether there are racial differences in brain RAS function, it is important to address these mechanistic questions in all ethnic groups. Estrogen also has a role in RAS-mediated effects on AD pathology, while differences across race in genetic coding for drug-metabolizing enzymes and transporters suggest that these effects could vary across sex and race/ethnicity [31, 32]. Some medications for chronic conditions, such as statins, have differential associations with AD risk across subpopulations, but such relationships have not been similarly examined for RAS-acting and non-RAS-acting AHTs [33].

To our knowledge, this is the first study to examine AD risk reduction among six different anti-hypertensive medications including both RAS-acting and non-RAS-acting AHTs in African American, Hispanic, and Caucasian men and women. While the different AHTs are similarly effective in reducing blood pressure, differences in their association with AD risk for black, non-Hispanic white, and white men and women could point to effects independent of blood pressure and have implications for clinicians, particularly those who serve minority populations [34, 35]. We used longitudinal claims data from 2007–2013 for a representative sample of fee-for-service Medicare beneficiaries ages 67 and older to analyze the association of RAS-acting AHT use compared to non-RAS-acting AHT use and AD onset. We examined differences within RAS-acting AHTs by comparing ACEIs and ARBs, and whether associations vary by sex and race/ethnicity in multivariable regressions that control for age, sex, race, education, income, comorbidities, health care utilization tendencies, and statin use.

## Methods

### Data

We examined the medical and pharmacy claims of a random 20% sample of Medicare beneficiaries enrolled in traditional Medicare (fee-for-service) from 2007 to 2013. The enrollment file includes beneficiary characteristics, which can be linked with claims from Medicare Parts A, B, and D. Part D claims include key elements related to prescription drug events, while Parts A and B claims capture inpatient and outpatient encounters, including detailed diagnosis and procedure codes (*International Classification of Diseases*, *Ninth Revision* (ICD-9)). These data were further supplemented with claims histories from the Chronic Conditions Warehouse. Institutional review board approval was granted by the University of Southern California University Park IRB, which granted a waiver of participant consent under 45 CFR 46.116(d).

### Study sample

The study sample consisted of Medicare beneficiaries age 67 and older. We required observation of each individual for a minimum of three years with consecutive fee-for-service enrollment, Part D enrollment, and no death. Individuals were required to have used at least one AHT class for two consecutive years, no prior AD diagnoses, and no prior use of acetylcholinesterase inhibitors (AChEIs) or memantine. We required an AD index diagnosis (ICD-9 331.0) to be verified with either a second AD diagnosis code or ‘dementia specified elsewhere’ diagnosis code in a subsequent claim within the study period (2009–2013). The analytic sample consisted of 1,343,334 unique beneficiaries, followed for a total of 4,215,338 person-years (2,820,575 for females, 3,501,668 for non-Hispanic whites, 295,521 for blacks, 230,887 for Hispanics, and 187,262 for Asian, Native American, or unknown race/ethnicity). Table 1 shows the characteristics of the analytic sample.

**Table 1:** Sample characteristics.

	Any AHT	RAS	no RAS	ACEI	ARB	Other 4
AD (verified)	0.97%	0.88%	1.12%	0.92%	0.81%	0.97%
# of AHT classes used	2.1	2.5	1.5	2.4	2.6	2.3
Combo use rate	69%	84%	42%	83%	87%	79%
Age	78.3	78.0	78.9	77.8	78.2	78.4
Female	67%	66%	69%	62%	72%	68%
White	83%	82%	85%	84%	79%	83%
Black	7%	7%	7%	7%	7%	7%
Hispanic	5%	6%	4%	6%	7%	5%
Other	4%	5%	4%	3%	7%	4%
% HS grad	76%	75%	76%	75%	75%	75%
Median income	$54,794	$54,777	$54,826	$53,846	$56,252	$54,577
# physician visits	9.4	9.3	9.5	8.8	10.4	9.5
Comorbidity index	1.34	1.31	1.41	1.31	1.31	1.37
Percent of sample with prior diagnosis of:						
AMI	7%	7%	7%	7%	6%	7%
ATF	20%	18%	23%	18%	18%	21%
Diabetes	44%	47%	36%	47%	48%	44%
Stroke	18%	17%	19%	17%	17%	18%
Hyperlipidemia	88%	89%	85%	88%	91%	88%
Non-AD dementia	8%	8%	10%	8%	7%	8%
Person-years	4,215,338	2,727,821	1,487,517	1,764,546	1,034,502	3,708,796
Unique beneficiaries	1,343,334	942,456	615,116	640,715	380,606	1,204,168

Sample characteristics of 2009–2013 Medicare enrollees with use of six classes of antihypertensive (AHT) prescription drugs (two renin-angiotensin (RAS) acting classes: angiotensin converting enzyme inhibitors (ACEIs) and angiotensin-II receptor blockers (ARBs), and four other classes: beta-blockers, calcium channel blockers, loop diuretics, and thiazide diuretics). Users defined as those with 90 possession days and 2 claims in year t-1 and year t-2. Sample restricted to person-years with 3 years fee-for-service, 3 years Part D, age 67+, no deaths in the reference year (year t), no prior AD diagnoses, and no prior use of acetylcholinesterase inhibitors (AChEIs) or memantine. The comorbidity index is the Hierarchical Condition Category (HCC) risk adjuster.

### Measuring AHT exposure

**Fig 1:**
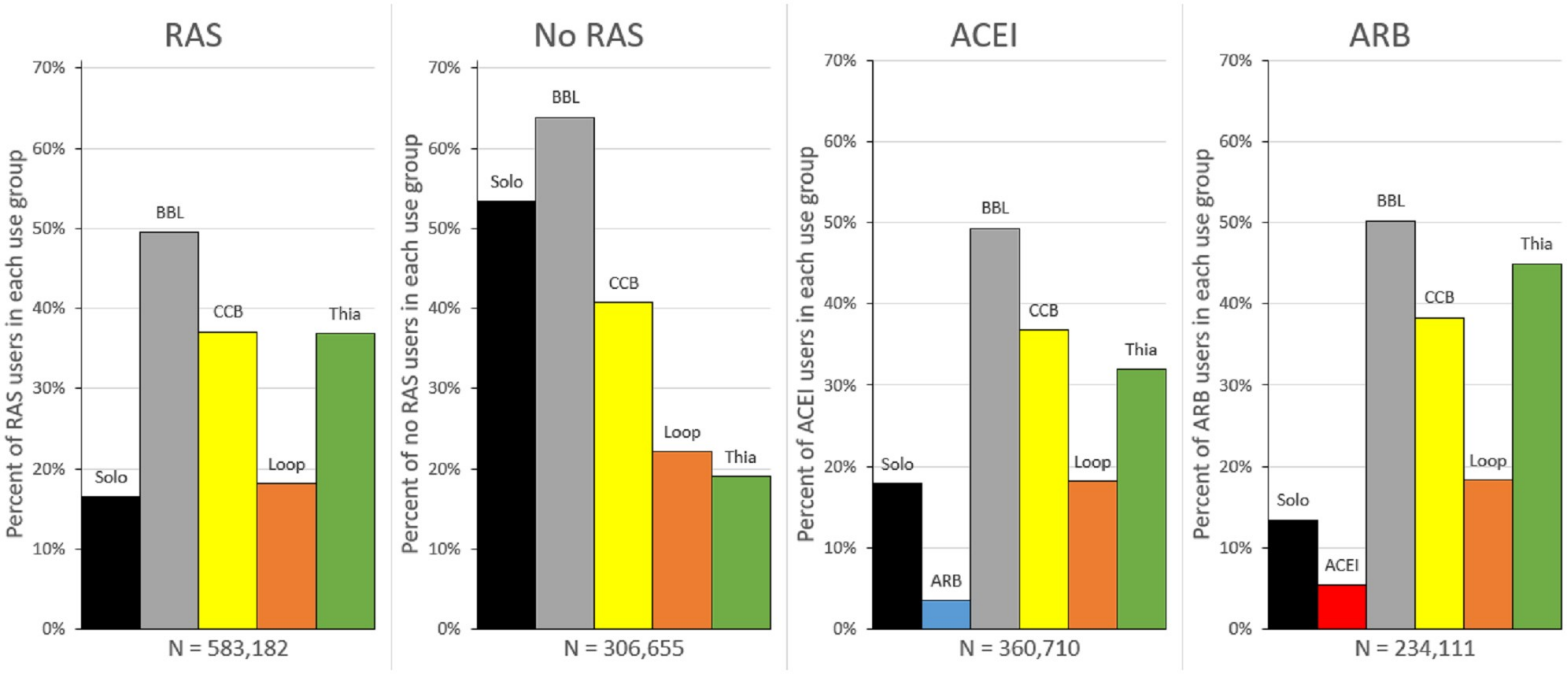
Antihypertensive (AHT) use groups in 2013. 2013 use of AHTs by Medicare enrollees (angiotensin converting enzyme inhibitors (ACEIs), angiotensin-II receptor blockers (ARBs), beta-blockers (BBL), calcium channel blockers (CCB), loop diuretics, and thiazide diuretics). Users defined as those with 90 possession days and 2 claims. Sample restricted to 2013 observations with sample restrictions described in Table 1. RAS (renin angiotensin system) acting drugs are ACEIs and ARBs.

We identified AHT use by selecting Part D claims (2007–2013) for the following classes: angiotensin-converting enzyme inhibitors (ACEI), angiotensin-II receptor blockers (ARB), beta-blockers (BBL), calcium channel blockers (CCB), loop diuretics (LDs), and thiazide-like diuretics (TDs). We defined an AHT user as any individual with 90 days supply and at least two drug claims in a year for two consecutive years. This threshold was chosen as the minimum necessary to ensure regular use of the drug during the exposure period, but other definitions were used in sensitivity analyses (see Limitations, below). Combination use of multiple AHTs is relatively common; Fig 1 shows the percent of the study sample using each of the AHT drug classes in 2013. Participants were classified as RAS-acting AHT users if they were taking a RAS AHT drug alone, or in combination with another AHT.

### Study design

We compared onset of AD for RAS-acting AHT drug users to those using only non-RAS-acting AHTs, using index date of AD diagnosis. Measurement error in the timing of AD diagnosis does is unlikely to bias our results because the same error exists for the AHT users in both the exposure and comparator groups. To mitigate concern that imminent AD onset could lead to poor adherence or discontinuation of AHT use, we designated years *t-1* and *t-2* as AHT exposure years, prior to assessing AD risk in year *t*. In addition to analyses that define RAS use as the aggregate of ACEI and ARB, we also compared the independent effects of ACEI and ARB use to non-RAS-acting AHT use. Sex and race/ethnicity specific analyses use only members of the same subgroup as the comparison group, thus estimated differences in association with type of AHT is attributed to drug class and not due to (unobserved) differences across race/ethnicity.

### Statistical analyses

We examined the association of RAS AHT use (in years *t-1* and *t-2*) and incident AD (year *t*), during the years *t* = 2009 to *t* = 2013. We used multivariable logistic regression to control for the potentially confounding roles of age, age squared, sex, race, high school graduation rate within the beneficiary’s zip code (quartiles), zip code median income (quartiles), statin use, years since hypertension diagnosis, comorbidity index (quartiles), number of physician visits (quartiles), and indicators for past diagnoses of diabetes, atrial fibrillation, acute myocardial infarction, stroke, and hyperlipidemia. Time-varying covariates were measured in year *t-1*. Health status was measured with past diagnosis of key comorbidities, as well as the Centers for Medicaid and Medicare Services-Hierarchical Condition Category (CMS-HCC), an index based on health status from diagnostic data and demographics, in which higher numbers indicate worse health. The CMS uses this index to predict health expenditures in the next year, and it correlates highly with mortality [36]. We used years since hypertension diagnosis, as measured by the Chronic Conditions Warehouse (CCW), to control for unobserved AHT use in the years prior to Medicare Part D enrollment. Race/ethnicity was determined with the beneficiary race code in CMS enrollment data, and with the application of a name-based identification algorithm from the Research Triangle Institute [37]. Sex and race/ethnicity specific analyses use only members of the same subgroup as the comparator. Standard errors were clustered at the county level. We ran analyses for the sample as a whole, as well as for each sex-race/ethnicity subgroup.

## Results

Table 1 shows the sample characteristics by type of AHT. The average age of RAS users is 78.0 years, while those using the other four AHTs averaged 78.4 years. RAS users had an average comorbidity score of 1.31 and averaged 9.3 physician visits per year, compared to 1.37 and 9.5 for users of the other four non-RAS-acting AHTs. Comorbidity prevalence across the two groups was: acute myocardial infarction (7% for both groups), atrial fibrillation (18% for RAS users, 21% for other non-RAS-acting AHT users), diabetes (47% for RAS users, 44% for non-RAS-acting AHT users), stroke (17% for RAS users, 18% for non-RAS-acting AHT users), hyperlipidemia (89% for RAS users, 88% for non-RAS-acting AHT users), and non-AD dementia (8% for both groups). Clinical guidelines currently recommend both RAS-acting and non-RAS-acting AHTs as first line therapies for hypertension, in the nonblack hypertensive population, including those with diabetes. Notably, 2017 blood pressure guidelines advise that initial AHT treatment for African Americans should include a TD or CCB instead of a RAS-acting AHT [35].

During the years 2009–2013, 0.97% of AHT users were diagnosed with AD each year (Table 1). RAS-acting AHT users had statistically significantly lower rates of AD incidence (0.88%), compared to non-RAS-acting AHT users (0.97%). ARB users had a statistically significantly lower incidence rate (0.81%), compared to ACEI users (0.92%). For comparison, in Medicare samples without drug use or death restrictions, we find AD incidence rates between 2.0% and 2.7%, depending on how restrictive we are in determining who is at risk. The Alzheimer’s Association estimates that the general population ages 65 and older had an AD incidence rate of 1.15% in 2013. [38, 39]

**Fig 2:**
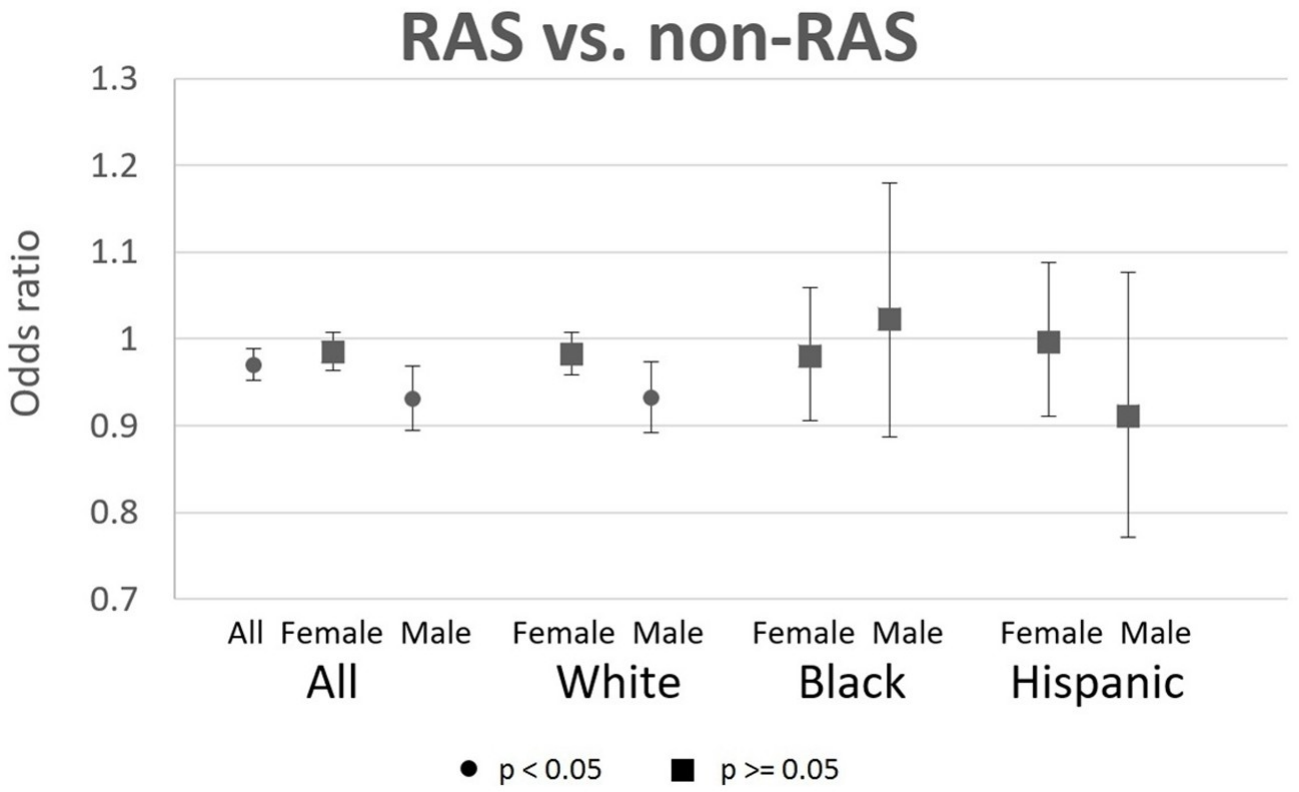
Odds ratios of AD incidence for RAS users relative to non-RAS users, with 95% CIs. Each OR is the result from a separate logistic regression, which compares RAS AHT users to non-RAS AHT users in the same sex-race/ethnicity subgroup. Sample of 2009–2013 Medicare enrollees with use of antihypertensive (AHT) prescription drugs (angiotensin converting enzyme inhibitors (ACEIs), angiotensin-II receptor blockers (ARBs), beta-blockers, calcium channel blockers, loop diuretics, and thiazide diuretics). Sample sizes of person-years are: 4215338 for all, 2820575 for females, 1394763 for males, 2324640 for white females, 1177028 for white males, 221664 for black females, 73857 for black males, 154142 for Hispanic females, and 76745 for Hispanic males. Users defined as those with 90 possession days and 2 claims in year t-1 and year t-2. Sample restricted to person-years with 3 years fee-for-service, 3 years Part D, age 67+, no deaths in the reference year (year t), no prior AD diagnoses, and no prior use of acetylcholinesterase inhibitors (AChEIs) or memantine. RAS (renin angiotensin system) acting drugs are ACEIs and ARBs. Controls are age, age squared, sex, race, education, income quartiles, statin use (t-1), years since hypertension diagnosis, HCC comorbidity index, number of physician visits, and indicators for past diagnoses of diabetes, atrial fibrillation, acute myocardial infarction, stroke, and hyperlipidemia. Standard errors are clustered at the county level.

The odds ratios (OR) from multivariable logistic regressions of AD risk and RAS-acting AHT use are depicted in Fig 2, with odds ratios, p-values, and confidence intervals provided in S1 Table. Each OR is the result from a separate regression, which compares RAS AHT users to non-RAS AHT users in the same sex-race/ethnicity subgroup. Use of RAS AHTs was associated with significantly reduced risk of AD for males (OR = 0.931, CI: 0.895–0.969), but not females (OR = 0.985, CI: 0.963–1.007). The significant association for males was largely driven by white males (OR = 0.932, CI: 0.892–0.974). Black and Hispanic males, and females of all races/ethnicities had OR close to 1.0 and no significant association between RAS AHT use and AD risk. Hispanic males (OR = 0.911, CI: 0.771–1.076) showed no significant association driven by large confidence intervals.

**Fig 3:**
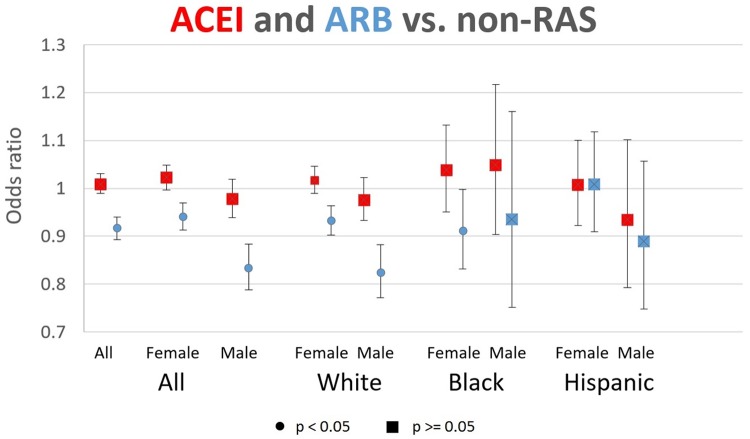
Odds ratios of AD incidence for ACEI and ARB users, relative to non-RAS users, with 95% Cis. Each pair of ORs (for ACEI and ARB) represents results from a separate logistic regression, which compares ACEI and ARB users to non-RAS users in the same sex-race/ethnicity subgroup. Sample is 2009–2013 Medicare enrollees with use of antihypertensive (AHT) prescription drugs (angiotensin converting enzyme inhibitors (ACEIs), angiotensin-II receptor blockers (ARBs), beta-blockers, calcium channel blockers, loop diuretics, and thiazide diuretics). Sample sizes of person-years are: 4215338 for all, 2820575 for females, 1394763 for males, 2324640 for white females, 1177028 for white males, 221664 for black females, 73857 for black males, 154142 for Hispanic females, and 76745 for Hispanic males. Users defined as those with 90 possession days and 2 claims in year t-1 and year t-2. Sample restricted to person-years with 3 years fee-for-service, 3 years Part D, age 67+, no deaths in the reference year (year t), no prior AD diagnoses, and no prior use of acetylcholinesterase inhibitors (AChEIs) or memantine. RAS (renin angiotensin system) acting drugs are ACEIs and ARBs. Controls are age, age squared, sex, race, education, income quartiles, statin use (t-1), years since hypertension diagnosis, HCC comorbidity index, number of physician visits, and indicators for past diagnoses of diabetes, atrial fibrillation, acute myocardial infarction, stroke, and hyperlipidemia. Standard errors are clustered at the county level.

Results from models that separated RAS AHTs into their component classes (ACEIs and ARBs) are reported in Fig 3 and S2 Table. Each pair of ORs (for ACEI and ARB) represents results from a separate regression, which compares ACEI and ARB users to non-RAS users in the same sex-race/ethnicity subgroup. The use of ACEIs had no significant association with AD risk, across men and women and all races/ethnicities. By contrast, ARB use showed significant protection against AD for white females (OR = 0.933, CI: 0.902–0.964), black females (OR = 0.912, CI: 0.832–0.998), and white males (OR = 0.825, CI: 0.772–0.882).

## Discussion

We analyzed the association between use of RAS-acting AHTs and AD onset across sex and race/ethnicity, and allowed for the possible separate biological roles of ACEIs and ARBs as has been suggested for AD [18, 40, 41]. While there is some evidence of risk reduction from RAS-acting AHTs, most of that association is driven by ARBs rather than ACEI use.

Why RAS AHTs may provide benefit in AD, compared to other AHTs, is potentially driven by a combination of genetic, pre-clinical, epidemiological, and a limited number of directly relevant clinical factors [18, 42, 43]. Multiple components of the RAS are associated with changes to amyloid-beta (Aβ) and tau levels in both pre-clinical models as well as human post-mortem studies [18, 24, 44–46]. Furthermore, RAS-acting AHTs have been reported to reduce the incidence of AD and slow the progression from MCI to AD in African Americans and Caucasians, leading to new studies whereby RAS-acting drugs are being tested as interventions for AD [12, 18, 19, 40, 41, 47].

Both ARBs and ACEIs were designed to treat hypertension by reducing signaling of the angiotensin II peptide hormone via the angiotensin II type-1 receptor (AT1R), but they differ in how they achieve the reduction in angiotensin II, and may differentially affect processes related to AD [3, 27]. ACEIs inhibit the activity of angiotensin I-converting enzyme (ACE) and thus the formation of angiotensin II. ARBs do not directly interfere with angiotensin II production, but instead block the binding and signaling of angiotensin II via its receptor AT1R. However, ACE has also been reported to degrade Aβ, and it has therefore been suggested that ACEIs may adversely alter ACE-mediated degradation of Aβ in the brain [27]. This would likely be disadvantageous, since Aβ is thought to be a significant factor in the development and pathology of AD. Yet ARBs could selectively inhibit the detrimental effects of angiotensin II without inhibiting ACE.

The potential involvement of the RAS in AD is particularly important in the African American population, because of differences in how the RAS functions in this ethnic group. The peripheral RAS functions differently between African Americans and Caucasians, with higher endogenous sodium and lower renin levels for African Americans. It is less clear, however, if in the brain, RAS functions differently between groups, but data suggest that AD related brain benefits via RAS therapy are applicable to both groups [12]. Additionally, both AD incidence and prevalence are higher in African Americans compared to Caucasians [48]. These results are especially important because the US population aged 65 years and older is projected to more than double by 2060 [49]. Over the same time period, the African American population is expected to grow from 14% to 18% of the total population, while the single race non-Hispanic white population is expected to decrease from 62% to 44% [50]. African Americans are also more likely to be hypertensive, an independent risk factor for AD, leading to an overall less healthy population. Thus, understanding the potentially beneficial effects of certain RAS-acting AHTs in this high-risk population is of great importance.

The results show that RAS AHTs are associated with lower AD risk for males (OR = 0.931, CI: 0.895–0.969) than females (OR = 0.985, CI: 0.963–1.007). In models that separated ACEIs and ARBs, the level of risk lowering in the ARBs was greater for males (OR = 0.834, CI: 0.788–0.884) than females (OR = 0.941, CI: 0.913–0.969). These disparities could relate to interactions that exist between estrogen and the RAS throughout life that may also have a bearing on late life RAS-mediated changes to AD pathology [32]. It is also possible that the beneficial effects of RAS-acting AHTs would be more evident in younger and middle aged women who are nearer the menopausal transition, while RAS related benefits for men may extend across the lifespan. It is worth noting that RAS-acting antihypertensives are contraindicated during the second and third trimesters of pregnancy because of increased risk of fetal renal damage, so women taking RAS therapy would be prescribed different antihypertensives during this time. It is therefore possible that women who are planning to become pregnant change their antihypertensive regimen and are likely to stay on non-RAS medications after pregnancy. As such, the RAS benefits favoring men may reflect longer exposure times from RAS-acting medications favoring longer brain related protection.

Unlike the other population subgroups, there was no benefit on average of ARBs for Hispanic females (OR = 0.996, CI: 0.911–1.088). It is not clear why there was no benefit in this population, particularly when there is high prevalence of RAS-associated obesity, metabolic disorders, and hypertension, all of which are risk factors for AD [51]. Yet, very little is known regarding whether there are ethnic differences in the RAS in Hispanics, in the same way that differences exist between Caucasians and African Americans. Unlike Caucasians and African Americans, there are currently no widely disseminated guidelines for treating hypertension in Hispanic populations, likely because many clinical studies have not included Hispanic populations and there is little literature describing RAS function among Hispanics [51–53].

While there is now some evidence of long term effects of midlife hypertension management on improved late-life cognition, our study focuses on the role of relatively recent hypertension treatment for older adults [54]. This approach is driven by evidence suggesting that there are also short term effects of AHT treatment on cognition, especially for ARBs [19, 55]. Of note, our results showing cognitive preservation are likely the result of multiple RAS-related benefits, in addition to the medications’ direct action on tau and amyloid-β. Mechanisms include improved or preserved cerebral blood flow, anti-inflammatory effects, and pro-cholinergic effects [14, 25, 56, 57]. We have also reported that RAS-acting medications, used for only four months, were able to cross the blood brain barrier and alter ACE activity and levels in the brain in middle aged individuals with a parental history of AD [58]. This suggests that while the brain and peripheral RAS operate largely independently, so long as the blood brain barrier is not compromised, RAS-acting medications are able to change components of the brain RAS in a relatively short amount of time.

### Limitations

The Medicare claims data provide an excellent setting for this type of study, with large and diverse samples, and no recall bias. There are, however, several limitations to these data, including the possibility that AHT use and/or AD are measured imprecisely. For example, switching between classes of AHT within a year would allow someone to be marked as a user of multiple classes within a year, or have some uncaptured use. This behavior, however, would lessen the observed differences across classes, and therefore bias our results toward zero. To address concerns about the effects of AHT use that occurred prior to observation in 2007, we control for years since hypertension diagnosis, which is observed as far back as 1999 if continuously enrolled in FFS Medicare. We are also concerned that AD diagnosis practices are imprecise, which can lead to measurement error in the timing of diagnosis, and across types of dementias. To address these problems, we require that AD diagnoses are verified on subsequent claims. Additionally, this type of measurement error would only be a problem if it varied systematically across AHT classes, which seems implausible. Another limitation of the claims data is that we are unable to control for apolipoprotein E (ApoE) status given current clinical practice does not test for ApoE.

When comparing users of multiple classes of AHTs, it is possible that unobserved patient characteristics could sort users across classes, and also affect AD risk differences. Few observable differences were found (Table 1) and we include a detailed set of control variables such as, age, comorbid conditions, zip code level high school graduation rates, and zip-code level median income in our estimation models. Additionally, all six classes of AHTs have generic versions available within the years of our study, making them inexpensive for our sample of Medicare Part D beneficiaries, and therefore diminishing the potential for socioeconomic differences to influence drug choices. Ideally, we would be able to control for blood pressure, so that we could precisely identify the role of AHT classes, net of blood pressure effects. Since we cannot observe blood pressure, we include controls for comorbidities that serve as proxies for extreme blood pressure: acute myocardial infarction, atrial fibrillation, and stroke.

Differences in the guidelines for hypertension treatment across ethnic groups might also cause systematic variation in the characteristics of AHT users across class. However, any such treatment selection patterns would be held constant in our analyses of population subgroups, which only compare each sex and race/ethnicity to members of the same group. Additionally, Table 1 shows the characteristics of the different AHT use groups, and gives empirical evidence that these groups have similar underlying characteristics, suggesting that protectiveness differences across class are not driven by patient selection.

The study period investigated here coincided with rapid growth in enrollment in Medicare Advantage plans. This selective attrition would only potentially influence the results if users of a particular AHT class were more or less likely to join a Medicare Advantage plan, which is likely implausible. However, we conducted sensitivity analyses on a sample restricted to subjects that survived and were observed through to the end of 2013 (no movement between fee-for-service and Medicare Advantage plans). The results of these analyses are featured in S3 and S4 Tables, and show no meaningful differences from the main results.

As shown in Fig 1, use of combinations of AHTs within a single year is common. For this reason, differential combination patterns between ACEIs and ARBs could be responsible for the relatively greater AD protection from ARB use. Fig 1 shows relatively similar combination use patterns for these two classes, except for TD use that is more common for ARB users than ACEI users. Ordinarily there are certain considerations, due to drug interactions, of what types of diuretics can be taken with RAS-acting AHTs. As such, potassium sparing diuretics (which TDs are not) do not tend to be given together with RAS-acting drugs to reduce the risk of hyperkalemia [59]. To ensure that the results are not driven by the differences in TD use, we conducted a sensitivity analysis in a sample that dropped all observations with use of TDs. These results (S5 and S6 Tables) show small changes in the magnitudes of some of the estimates, but the not in the statistical significance and are consistent with the main results.

Other robustness checks included varying the definition of user, with thresholds at 180 (S7 and S8 Tables) and 270 (S9 and S10 Tables) days of use, instead of 90. These adjustments require more adherent use of all the AHTs, including the non-RAS-acting classes in our comparison group. These results show that the differences between ACEIs and ARBs, and across sex, are robust to the definition of user. Differences between RAS and non-RAS drugs for white males were no longer statistically different with a more restrictive definition of user (270 days).

## Conclusion

Our findings suggest that the use of certain AHTs could provide benefits in addition to those ordinarily associated with blood pressure reductions. ARBs may be more protective against AD than ACEIs, and thus could reduce some of the burden of AD for the afflicted individuals, their caregivers, and the health system as a whole. The burden of AD is multifaceted and disparate, and even small delays in AD onset can lead to disproportionately larger reductions in this large and growing burden. For example, if it were possible to achieve a one year delay in the onset of AD, this would lead to a reduction in the U.S. population ages 70 and older with AD in 2050 by 14% and savings of $219 billion in medical and caregiving costs in 2050 [2]. A five-year delay in AD onset would reduce the U.S. population with AD in 2050 by 41% [2].

## Supporting information

S1 Table Odds ratios (with 95% CI) of AD incidence associated with use of RAS AHTs, relative to non-RAS users. Each OR is the result from a separate regression, which compares RAS AHT users to non-RAS AHT users in the same sex-race/ethnicity subgroup. Logistic regression results with sample of 2009–2013 Medicare enrollees with use of antihypertensive (AHT) prescription drugs (angiotensin converting enzyme inhibitors (ACEIs), angiotensin-II receptor blockers (ARBs), beta-blockers, calcium channel blockers, loop diuretics, and thiazide diuretics). Users defined as those with 90 possession days and 2 claims in year t-1 and year t-2. Sample restricted to person-years with 3 years fee-for-service, 3 years Part D, age 67+, no deaths in the reference year (year t), no prior AD diagnoses, and no prior use of acetylcholinesterase inhibitors (AChEIs) or memantine. RAS (renin angiotensin system) acting drugs are ACEIs and ARBs. Controls are age, age squared, sex, race, education, income quartiles, statin use (t-1), years since hypertension diagnosis, HCC comorbidity index, number of physician visits, and indicators for past diagnoses of diabetes, atrial fibrillation, acute myocardial infarction, stroke, and hyperlipidemia. Standard errors are clustered at the county level.
(DOCX)

S2 Table Odds ratios (with 95% CI) of AD incidence associated with use of ACEI and ARBs, relative to non-RAS users. Each pair of ORs (for ACEI and ARB) represents results from a separate regression, which compares ACEI and ARB users to non-RAS users in the same sex-race/ethnicity subgroup. Logistic regression results with sample of 2009–2013 Medicare enrollees with use of antihypertensive (AHT) prescription drugs (angiotensin converting enzyme inhibitors (ACEIs), angiotensin-II receptor blockers (ARBs), beta-blockers, calcium channel blockers, loop diuretics, and thiazide diuretics). Users defined as those with 90 possession days and 2 claims in year t-1 and year t-2. Sample restricted to person-years with 3 years fee-for-service, 3 years Part D, age 67+, no deaths in the reference year (year t), no prior AD diagnoses, and no prior use of acetylcholinesterase inhibitors (AChEIs) or memantine. RAS (renin angiotensin system) acting drugs are ACEIs and ARBs. Controls are age, age squared, sex, race, education, income quartiles, statin use (t-1), years since hypertension diagnosis, HCC comorbidity index, number of physician visits, and indicators for past diagnoses of diabetes, atrial fibrillation, acute myocardial infarction, stroke, and hyperlipidemia. Standard errors are clustered at the county level.
(DOCX)

S3 Table Odds ratios (with 95% CI) of AD incidence associated with use of RAS AHTs, relative to non-RAS users, in sample with no attrition through 2013. Logistic regression results with sample of 2009–2013 Medicare enrollees with use of antihypertensive (AHT) prescription drugs (angiotensin converting enzyme inhibitors (ACEIs), angiotensin-II receptor blockers (ARBs), beta-blockers, calcium channel blockers, loop diuretics, and thiazide diuretics). Each OR is the result from a separate regression, which compares RAS AHT users to non-RAS AHT users in the same sex-race/ethnicity subgroup. Users defined as those with 90 possession days and 2 claims in year t-1 and year t-2. Sample restricted to person-years with 3 years fee-for-service, 3 years Part D, age 67+, no deaths in the reference year (year t), no prior AD diagnoses, no prior use of acetylcholinesterase inhibitors (AChEIs) or memantine, and no attrition through 2013 (except for AD diagnosis). RAS (renin angiotensin system) acting drugs are ACEIs and ARBs. Controls are age, age squared, sex, race, education, income quartiles, statin use (t-1), years since hypertension diagnosis, HCC comorbidity index, number of physician visits, and indicators for past diagnoses of diabetes, atrial fibrillation, acute myocardial infarction, stroke, and hyperlipidemia. Standard errors are clustered at the county level.
(DOCX)

S4 Table Odds ratios (with 95% CI) of AD incidence associated with use of ACEI and ARBs, relative to non-RAS users, in sample with no attrition through 2013. Logistic regression results with sample of 2009–2013 Medicare enrollees with use of antihypertensive (AHT) prescription drugs (angiotensin converting enzyme inhibitors (ACEIs), angiotensin-II receptor blockers (ARBs), beta-blockers, calcium channel blockers, loop diuretics, and thiazide diuretics). Each pair of ORs (for ACEI and ARB) represents results from a separate regression, which compares ACEI and ARB users to non-RAS users in the same sex-race/ethnicity subgroup. Users defined as those with 90 possession days and 2 claims in year t-1 and year t-2. Sample restricted to person-years with 3 years fee-for-service, 3 years Part D, age 67+, no deaths in the reference year (year t), no prior AD diagnoses, no prior use of acetylcholinesterase inhibitors (AChEIs) or memantine, and no attrition through 2013 (except for AD diagnosis). RAS (renin angiotensin system) acting drugs are ACEIs and ARBs. Controls are age, age squared, sex, race, education, income quartiles, statin use (t-1), years since hypertension diagnosis, HCC comorbidity index, number of physician visits, and indicators for past diagnoses of diabetes, atrial fibrillation, acute myocardial infarction, stroke, and hyperlipidemia. Standard errors are clustered at the county level.
(DOCX)

S5 Table Odds ratios (with 95% CI) of AD incidence associated with use of RAS AHTs, relative to non-RAS users, in sample with no use of thiazide-like diuretics. Logistic regression results with sample of 2009–2013 Medicare enrollees with use of antihypertensive (AHT) prescription drugs (angiotensin converting enzyme inhibitors (ACEIs), angiotensin-II receptor blockers (ARBs), beta-blockers, calcium channel blockers, loop diuretics, and thiazide diuretics). Each OR is the result from a separate regression, which compares RAS AHT users to non-RAS AHT users in the same sex-race/ethnicity subgroup. Users defined as those with 90 possession days and 2 claims in year t-1 and year t-2. Sample restricted to person-years with 3 years fee-for-service, 3 years Part D, age 67+, no deaths in the reference year (year t), no prior AD diagnoses, no prior use of acetylcholinesterase inhibitors (AChEIs) or memantine, and no use of thiazide-like diuretics. RAS (renin angiotensin system) acting drugs are ACEIs and ARBs. Controls are age, age squared, sex, race, education, income quartiles, statin use (t-1), years since hypertension diagnosis, HCC comorbidity index, number of physician visits, and indicators for past diagnoses of diabetes, atrial fibrillation, acute myocardial infarction, stroke, and hyperlipidemia. Standard errors are clustered at the county level.
(DOCX)

S6 Table Odds ratios (with 95% CI) of AD incidence associated with use of ACEI and ARBs, relative to non-RAS users, in sample with no use of thiazide-like diuretics. Logistic regression results with sample of 2009–2013 Medicare enrollees with use of antihypertensive (AHT) prescription drugs (angiotensin converting enzyme inhibitors (ACEIs), angiotensin-II receptor blockers (ARBs), beta-blockers, calcium channel blockers, loop diuretics, and thiazide diuretics). Each pair of ORs (for ACEI and ARB) represents results from a separate regression, which compares ACEI and ARB users to non-RAS users in the same sex-race/ethnicity subgroup. Users defined as those with 90 possession days and 2 claims in year t-1 and year t-2. Sample restricted to person-years with 3 years fee-for-service, 3 years Part D, age 67+, no deaths in the reference year (year t), no prior AD diagnoses, no prior use of acetylcholinesterase inhibitors (AChEIs) or memantine, and no use of thiazide-like diuretics. RAS (renin angiotensin system) acting drugs are ACEIs and ARBs. Controls are age, age squared, sex, race, education, income quartiles, statin use (t-1), years since hypertension diagnosis, HCC comorbidity index, number of physician visits, and indicators for past diagnoses of diabetes, atrial fibrillation, acute myocardial infarction, stroke, and hyperlipidemia. Standard errors are clustered at the county level.
(DOCX)

S7 Table Odds ratios (with 95% CI) of AD incidence associated with use of RAS AHTs, relative to non-RAS users, with users defined at 180 possession days. Logistic regression results with sample of 2009–2013 Medicare enrollees with use of antihypertensive (AHT) prescription drugs (angiotensin converting enzyme inhibitors (ACEIs), angiotensin-II receptor blockers (ARBs), beta-blockers, calcium channel blockers, loop diuretics, and thiazide diuretics). Each OR is the result from a separate regression, which compares RAS AHT users to non-RAS AHT users in the same sex-race/ethnicity subgroup. Users defined as those with 180 possession days and 2 claims in year t-1 and year t-2. Sample restricted to person-years with 3 years fee-for-service, 3 years Part D, age 67+, no deaths in the reference year (year t), no prior AD diagnoses, and no prior use of acetylcholinesterase inhibitors (AChEIs) or memantine. RAS (renin angiotensin system) acting drugs are ACEIs and ARBs. Controls are age, age squared, sex, race, education, income quartiles, statin use (t-1), years since hypertension diagnosis, HCC comorbidity index, number of physician visits, and indicators for past diagnoses of diabetes, atrial fibrillation, acute myocardial infarction, stroke, and hyperlipidemia. Standard errors are clustered at the county level.
(DOCX)

S8 Table Odds ratios (with 95% CI) of AD incidence associated with use of ACEI and ARBs, relative to non-RAS users, with users defined at 180 possession days. Logistic regression results with sample of 2009–2013 Medicare enrollees with use of antihypertensive (AHT) prescription drugs (angiotensin converting enzyme inhibitors (ACEIs), angiotensin-II receptor blockers (ARBs), beta-blockers, calcium channel blockers, loop diuretics, and thiazide diuretics). Each pair of ORs (for ACEI and ARB) represents results from a separate regression, which compares ACEI and ARB users to non-RAS users in the same sex-race/ethnicity subgroup. Users defined as those with 180 possession days and 2 claims in year t-1 and year t-2. Sample restricted to person-years with 3 years fee-for-service, 3 years Part D, age 67+, no deaths in the reference year (year t), no prior AD diagnoses, and no prior use of acetylcholinesterase inhibitors (AChEIs) or memantine. RAS (renin angiotensin system) acting drugs are ACEIs and ARBs. Controls are age, age squared, sex, race, education, income quartiles, statin use (t-1), years since hypertension diagnosis, HCC comorbidity index, number of physician visits, and indicators for past diagnoses of diabetes, atrial fibrillation, acute myocardial infarction, stroke, and hyperlipidemia. Standard errors are clustered at the county level.
(DOCX)

S9 Table Odds ratios (with 95% CI) of AD incidence associated with use of RAS AHTs, relative to non-users, with users defined at 270 possession days. Logistic regression results with sample of 2009–2013 Medicare enrollees with use of antihypertensive (AHT) prescription drugs (angiotensin converting enzyme inhibitors (ACEIs), angiotensin-II receptor blockers (ARBs), beta-blockers, calcium channel blockers, loop diuretics, and thiazide diuretics). Each OR is the result from a separate regression, which compares RAS AHT users to non-RAS AHT users in the same sex-race/ethnicity subgroup. Users defined as those with 270 possession days and 2 claims in year t-1 and year t-2. Sample restricted to person-years with 3 years fee-for-service, 3 years Part D, age 67+, no deaths in the reference year (year t), no prior AD diagnoses, and no prior use of acetylcholinesterase inhibitors (AChEIs) or memantine. RAS (renin angiotensin system) acting drugs are ACEIs and ARBs. Controls are age, age squared, sex, race, education, income quartiles, statin use (t-1), years since hypertension diagnosis, HCC comorbidity index, number of physician visits, and indicators for past diagnoses of diabetes, atrial fibrillation, acute myocardial infarction, stroke, and hyperlipidemia. Standard errors are clustered at the county level.
(DOCX)

S10 Table Odds ratios (with 95% CI) of AD incidence associated with use of ACEI and ARBs, relative to non-users, with users defined at 270 possession days. Logistic regression results with sample of 2009–2013 Medicare enrollees with use of antihypertensive (AHT) prescription drugs (angiotensin converting enzyme inhibitors (ACEIs), angiotensin-II receptor blockers (ARBs), beta-blockers, calcium channel blockers, loop diuretics, and thiazide diuretics). Each pair of ORs (for ACEI and ARB) represents results from a separate regression, which compares ACEI and ARB users to non-RAS users in the same sex-race/ethnicity subgroup. Users defined as those with 270 possession days and 2 claims in year t-1 and year t-2. Sample restricted to person-years with 3 years fee-for-service, 3 years Part D, age 67+, no deaths in the reference year (year t), no prior AD diagnoses, and no prior use of acetylcholinesterase inhibitors (AChEIs) or memantine. RAS (renin angiotensin system) acting drugs are ACEIs and ARBs. Controls are age, age squared, sex, race, education, income quartiles, statin use (t-1), years since hypertension diagnosis, HCC comorbidity index, number of physician visits, and indicators for past diagnoses of diabetes, atrial fibrillation, acute myocardial infarction, stroke, and hyperlipidemia. Standard errors are clustered at the county level.
(DOCX)

## References

[1] Alzheimer’s Association, 2017 Alzheimer’s disease facts and figures. Alzheimer’s & Dementia, 2017 13(4): p. 325–373. 10.1016/j.jalz.2016.03.001 27570871

[2] Zissimopoulos J., Crimmins E., and St Clair P., The Value of Delaying Alzheimer’s Disease Onset. Forum for Health Economics and Policy, 2015 18(1): p. 25–39. 10.1515/fhep-2014-0013 PMC4851168 27134606

[3] Rouch L., Cestac P., Hanon O., Cool C., Helmer C., Bouhanick B., et al, Antihypertensive drugs, prevention of cognitive decline and dementia: a systematic review of observational studies, randomized controlled trials and meta-analyses, with discussion of potential mechanisms. CNS drugs, 2015 29(2): p. 113–130. 10.1007/s40263-015-0230-6 25700645

[4] Merai R., CDC grand rounds: a public health approach to detect and control hypertension. MMWR. Morbidity and mortality weekly report, 2016 65. 10.15585/mmwr.mm6545a3 27855138

[5] Go A.S., Mozaffarian D., Roger V.L., Benjamin E.J., Berry J.D., Blaha M., et al, Heart disease and stroke statistics—2014 update: a report from the American Heart Association. circulation, 2014 129(3): p. e28 10.1161/01.cir.0000441139.02102.80 24352519 PMC5408159

[6] *Health, United States, 2016: With Chartbook on Long-term Trends in Health*. 2017, National Center for Health Statistics: Hyatsville, MD. 28910066

[7] Marpillat N.L., Macquin-Mavier I., Tropeano A., Bachoud-Levi A., and Maison P., Antihypertensive classes, cognitive decline and incidence of dementia: a network meta-analysis. Journal of hypertension, 2013 31(6): p. 1073–1082. 10.1097/HJH.0b013e3283603f53 23552124

[8] Peters R., Beckett N., Forette F., Tuomilehto J., Clarke R., Ritchee C., et al, Incident dementia and blood pressure lowering in the Hypertension in the Very Elderly Trial cognitive function assessment (HYVET-COG): a double-blind, placebo controlled trial. The Lancet Neurology, 2008 7(8): p. 683–689. 10.1016/S1474-4422(08)70143-1 18614402

[9] McGuinness B., Todd S., Passmore P., and Bullock R., Blood pressure lowering in patients without prior cerebrovascular disease for prevention of cognitive impairment and dementia. Cochrane Database Syst Rev, 2006 2. 10.1002/14651858.CD004034.pub2 16625595

[10] Forette F., Seux M., Staessen J.A., Thijs L., Babarskiene M., Babeanu S., et al, The prevention of dementia with antihypertensive treatment: new evidence from the Systolic Hypertension in Europe (Syst-Eur) study. Archives of internal medicine, 2002 162(18): p. 2046–2052. 12374512 10.1001/archinte.162.18.2046

[11] Van Charante E.P.M., Richard E., Eurelings L.S., van Dalen J., Ligthart S.A., Van Bussel E.F., et al, Effectiveness of a 6-year multidomain vascular care intervention to prevent dementia (preDIVA): a cluster-randomised controlled trial. The Lancet, 2016 388(10046): p. 797–805. 10.1016/S0140-6736(16)30950-3 27474376

[12] Wharton W., Goldstein F.C., Zhao L., Steenland K., Levey A., and Hajjar I., Modulation of Renin-Angiotensin System May Slow Conversion from Mild Cognitive Impairment to Alzheimer’s Disease. Journal of the American Geriatrics Society, 2015 63(9): p. 1749–1756. 10.1111/jgs.13627 26389987 PMC4743657

[13] Raghavendra V., Chopra K., and Kulkarni S., Comparative studies on the memory-enhancing actions of captopril and losartan in mice using inhibitory shock avoidance paradigm. Neuropeptides, 2001 35(1): p. 65–69. 10.1054/npep.2000.0845 11346312

[14] Raghavendra V., Chopra K., and Kulkarni S., Involvement of cholinergic system in losartan-induced facilitation of spatial and short-term working memory. Neuropeptides, 1998 32(5): p. 417–421. 9845001 10.1016/s0143-4179(98)90065-8

[15] Fogari R., Mugellini A., Zoppi A., Derosa G., Pasotti C., Fogari E., et al, Influence of losartan and atenolol on memory function in very elderly hypertensive patients. Journal of human hypertension, 2003 17(11): p. 781–785. 10.1038/sj.jhh.1001613 14578918

[16] Tedesco M.A., Ratti G., Mennella S., Manzo G., Grieco M., Rainone A.C., et al, Comparison of losartan and hydrochlorothiazide on cognitive function and quality of life in hypertensive patients. American journal of hypertension, 1999 12(11): p. 1130–1134. 10604491 10.1016/s0895-7061(99)00156-9

[17] Poon I.O., Effects of antihypertensive drug treatment on the risk of dementia and cognitive impairment. Pharmacotherapy: The Journal of Human Pharmacology and Drug Therapy, 2008 28(3): p. 366–375. 10.1592/phco.28.3.366 18294116

[18] Kehoe P.G., The coming of age of the angiotensin hypothesis in Alzheimer’s disease—progress towards disease prevention and treatment? Journal of Alzheimer’s Disease, 2018 62(3). 10.3233/JAD-171119 PMC5870007 29562545

[19] Li N., Lee A., Whitmer R.A., Kivipelto M., Lawler E., Kazis L.E., et al, Use of angiotensin receptor blockers and risk of dementia in a predominantly male population: prospective cohort analysis. Bmj, 2010 340: p. b5465 10.1136/bmj.b5465 20068258 PMC2806632

[20] Anderson C., Teo K., Gao P., Arima H., Dans A., Unger T., et al, Renin-angiotensin system blockade and cognitive function in patients at high risk of cardiovascular disease: analysis of data from the ONTARGET and TRANSCEND studies. The Lancet Neurology, 2011 10(1): p. 43–53. 10.1016/S1474-4422(10)70250-7 20980201

[21] Diener H., Sacco R.L., Yusuf S., Cotton D., Ounpuu S., Lawton W.A., et al, Effects of aspirin plus extended-release dipyridamole versus clopidogrel and telmisartan on disability and cognitive function after recurrent stroke in patients with ischaemic stroke in the Prevention Regimen for Effectively Avoiding Second Strokes (PRoFESS) trial: a double-blind, active and placebo-controlled study. The Lancet Neurology, 2008 7(10): p. 875–884. 10.1016/S1474-4422(08)70198-4 18757238 PMC2772657

[22] Wharton, W., Zhao, L., Steenland, K., Gearing, M., and Goldstein, F.C., *Less Conversion to AD and Fewer Neurofibrillary Tangles in MCI Patients Taking Certain Antihypertensives*, in *Alzheimer’s Association International Conference*. 2017: London, UK.

[23] Wharton, W., Zhao, L., Steenland, K., Gearing, M., and Goldstein, F., *Fewer Neurofibrillary Tangles and Slower Conversion to AD in MCI Patients Taking RAS Acting Antihypertensives* in *Americans Neurological Association Conference*. 2016. Baltimore, MD.

[24] Hajjar I., Brown L., Mack W., and Chui H., Impact of Angiotensin receptor blockers on Alzheimer disease neuropathology in a large brain autopsy series. Archives of neurology, 2012 69(12): p. 1632–1638. 10.1001/archneurol.2012.1010 22964777 PMC3608189

[25] Hajjar I., Hart M., Chen Y., Mack W., Novack V., Chui H.C., et al, Antihypertensive therapy and cerebral hemodynamics in executive mild cognitive impairment: results of a pilot randomized clinical trial. Journal of the American Geriatrics Society, 2013 61(2): p. 194–201. 10.1111/jgs.12100 23350899 PMC3608194

[26] Hu J., Igarashi A., Kamata M., and Nakagawa H., Angiotensin-converting enzyme degrades Alzheimer amyloid β-peptide (Aβ); retards Aβ aggregation, deposition, fibril formation; and inhibits cytotoxicity. Journal of Biological Chemistry, 2001 276(51): p. 47863–47868. 10.1074/jbc.M104068200 11604391

[27] Hemming M.L. and Selkoe D.J., Amyloid β-protein is degraded by cellular angiotensin-converting enzyme (ACE) and elevated by an ACE inhibitor. Journal of Biological Chemistry, 2005 280(45): p. 37644–37650. 10.1074/jbc.M508460200 16154999 PMC2409196

[28] Zou K., Yamaguchi H., Akatsu H., Sakamoto T., Ko M., Mizoguchi K., et al, Angiotensin-converting enzyme converts amyloid β-protein 1–42 (Aβ1–42) to Aβ1–40, and its inhibition enhances brain Aβ deposition. Journal of Neuroscience, 2007 27(32): p. 8628–8635. 10.1523/JNEUROSCI.1549-07.2007 17687040 PMC6672927

[29] Kehoe P.G. and Wilcock G.K., Is inhibition of the renin–angiotensin system a new treatment option for Alzheimer’s disease? The Lancet Neurology, 2007 6(4): p. 373–378. 10.1016/S1474-4422(07)70077-7 17362841

[30] Kehoe P.G., Miners S., and Love S., Angiotensins in Alzheimer’s disease–friend or foe? Trends in neurosciences, 2009 32(12): p. 619–628. 10.1016/j.tins.2009.07.006 19796831

[31] Mangravite L., Thorn C., and Krauss R., Clinical implications of pharmacogenomics of statin treatment. The pharmacogenomics journal, 2006 6(6): p. 360–360. 10.1038/sj.tpj.6500384 16550210

[32] O’Hagan T.S., Wharton W., and Kehoe P.G., Interactions between oestrogen and the renin angiotensin system-potential mechanisms for gender differences in Alzheimer’s disease. American journal of neurodegenerative disease, 2012 1(3): p. 266 23383397 PMC3560469

[33] Zissimopoulos J.M., Barthold D., Brinton R.D., and Joyce G., Sex and Race Differences in the Association Between Statin Use and the Incidence of Alzheimer Disease. JAMA Neurology, 2016. 10.1001/jamaneurol.2016.3783 PMC5646357 27942728

[34] Law M.R., Wald N.J., Morris J.K., and Jordan R.E., Value of low dose combination treatment with blood pressure lowering drugs: analysis of 354 randomised trials. Bmj, 2003 326(7404): p. 1427 10.1136/bmj.326.7404.1427 12829555 PMC162261

[35] Cifu A.S. and Davis A.M., Prevention, detection, evaluation, and management of high blood pressure in adults. JAMA, 2017. 10.1001/jama.2017.18706 29159416

[36] Li P., Kim M.M., and Doshi J.A., Comparison of the performance of the CMS Hierarchical Condition Category (CMS-HCC) risk adjuster with the Charlson and Elixhauser comorbidity measures in predicting mortality. BMC health services research, 2010 10(1): p. 1. 20727154 10.1186/1472-6963-10-245 PMC2936901

[37] Bonito A., Bann C., Eicheldinger C., and Carpenter L., *Creation of new race-ethnicity codes and socioeconomic status (SES) indicators for Medicare beneficiaries*. 2008, Agency for Healthcare Research and Quality, and Center for Medicare and Medicaid Services: Rockville, MD.

[38] Alzheimer’s Association, 2018 Alzheimer’s disease facts and figures. Alzheimer’s & Dementia, 2018 14(3): p. 367–429.

[39] U.S. Department of Commerce, *Projected Age Groups and Sex Composition of the Population*: *Main Projection Series for the United States*, *2017–2060*, U.S. Dept of Commerce, Editor. 2017, U.S. Census Bureau, Population Division: Washington, DC.

[40] Wharton W., Goldstein F.C., Tansey M.G., Brown A.L., Tharwani S.D., Verble D.D., et al, Rationale and Design of the Mechanistic Potential of Antihypertensives in Preclinical Alzheimer’s (HEART) Trial. Journal of Alzheimer’s disease: JAD, 2018 61(2): p. 815 10.3233/JAD-161198 29254080 PMC8933850

[41] Kehoe P.G., Blair P.S., Howden B., Thomas D.L., Malone I.B., Horwood J., et al, The Rationale and Design of the Reducing Pathology in Alzheimer’s Disease through Angiotensin TaRgeting (RADAR) Trial. Journal of Alzheimer’s disease: JAD, 2018 61(2): p. 803 10.3233/JAD-170101 29226862

[42] de Oliveira F.F., Chen E.S., smith M.C., and Bertolucci P.H.F., Associations of Blood Pressure with Functional and Cognitive Changes in Patients with Alzheimer’s Disease. Dementia and geriatric cognitive disorders, 2016 41(5–6): p. 314–323. 10.1159/000447585 27398980

[43] de Oliveira F.F., Chen E.S., smith M.C., and Bertolucci P.H.F., Pharmacogenetics of angiotensin-converting enzyme inhibitors in patients with Alzheimer’s disease dementia. Current Alzheimer research, 2017. 10.2174/1567205014666171016101816 29034839

[44] Hou D., Wang Y., Zhou L., Chen K., Tian Y., Song Z., et al, Altered angiotensin-converting enzyme and its effects on the brain in a rat model of Alzheimer disease. Chinese medical journal, 2008 121(22): p. 2320–2323. 19080340

[45] Jiang T., Zhang Y., Zhou J., Zhu X., Tian Y., Zhao H., et al, Angiotensin-(1–7) is reduced and inversely correlates with Tau hyperphosphorylation in animal models of Alzheimer’s disease. Molecular neurobiology, 2016 53(4): p. 2489–2497. 10.1007/s12035-015-9260-9 26044748

[46] Kehoe P.G., Wong S., Mulhim N.A.L., Palmer L.E., and Miners J.S., Angiotensin-converting enzyme 2 is reduced in Alzheimer’s disease in association with increasing amyloid-β and tau pathology. Alzheimer’s research & therapy, 2016 8(1): p. 50. 10.1186/s13195-016-0217-7 PMC5123239 27884212

[47] Davies N.M., Kehoe P.G., Ben-Shlomo Y., and Martin R.M., Associations of anti-hypertensive treatments with Alzheimer’s disease, vascular dementia, and other dementias. Journal of Alzheimer’s Disease, 2011 26(4): p. 699–708. 10.3233/JAD-2011-110347 21709373

[48] Tang M., Cross P., Andrews H., Jacobs D.M., Small S., Bell K., et al, Incidence of AD in African-Americans, Caribbean hispanics, and caucasians in northern Manhattan. Neurology, 2001 56(1): p. 49–56. 11148235 10.1212/wnl.56.1.49

[49] Mather M., Jacobsen L.A., and Pollard K.M., Aging in the United States. Population Bulletin, 2015 70(2).

[50] Colby S.L. and Ortman J.M., Projections on the size and composition of the U.S. population: 2014 to 2060. Current Population Reports, 2014 P25–1143.

[51] Guzman N.J., Epidemiology and management of hypertension in the hispanic population. American Journal of Cardiovascular Drugs, 2012 12(3): p. 165–178. 10.2165/11631520-000000000-00000 22583147 PMC3624012

[52] Rifkin D.E., Khaki A.R., Jenny N.S., McClelland R.L., Budoff M., Watson K., et al, Association of renin and aldosterone with ethnicity and blood pressure: the multi-ethnic study of atherosclerosis. American journal of hypertension, 2014 27(6): p. 801–810. 10.1093/ajh/hpt276 24436325 PMC4017931

[53] Whelton P.K., Carey R.M., Aronow W.S., Casey D.E., Collins K.J., Himmelfarb C.D., et al, A guideline for the prevention, detection, evaluation, and management of high blood pressure in adults: a report of the American College of Cardiology/American Heart Association Task Force on Clinical Practice Guidelines. Journal of the American College of Cardiology, 2017: p. 24430. 10.1016/j.jacc.2017.11.006 29146535

[54] Livingston G., Sommerlad A., Orgeta V., Costafreda S.G., Huntley J., Ames D., et al, Dementia prevention, intervention, and care. The Lancet, 2017. 10.1016/S0140-6736(17)31363-6 28735855

[55] Hanon O., Berrou J., Negre-Pages L., Goch J.H., Nadhazi Z., Petrella R., et al, Effects of hypertension therapy based on eprosartan on systolic arterial blood pressure and cognitive function: primary results of the Observational Study on Cognitive function And Systolic Blood Pressure Reduction open-label study. Journal of hypertension, 2008 26(8): p. 1642–1650. 10.1097/HJH.0b013e328301a280 18622244

[56] Kumaran D., Udayabanu M., Kumar M., Aneja R., and Katyal A., Involvement of angiotensin converting enzyme in cerebral hypoperfusion induced anterograde memory impairment and cholinergic dysfunction in rats. Neuroscience, 2008 155(3): p. 626–639. 10.1016/j.neuroscience.2008.06.023 18621107

[57] Torika N., Asraf K., Apte R.N., and Fleisher-Berkovich S., Candesartan ameliorates brain inflammation associated with Alzheimer’s disease. CNS neuroscience & therapeutics, 2018 24(3): p. 231–242. 29365370 10.1111/cns.12802 PMC6489976

[58] Wharton W., Stein J.H., Korcarz C., Sachs J., Olson S.R., Zetterberg H., et al, The effects of ramipril in individuals at risk for Alzheimer’s disease: results of a pilot clinical trial. Journal of Alzheimer’s Disease, 2012 32(1): p. 147–156. 10.3233/JAD-2012-120763 22776970 PMC3593582

[59] Wrenger E., Muller R., Moesenthin M., Welte T., Frolich J.C., and Neumann K.H., Lesson of the week: Interaction of spironolactone with ACE inhibitors or angiotensin receptor blockers: analysis of 44 cases. BMJ: British Medical Journal, 2003 327(7407): p. 147 10.1136/bmj.327.7407.147 12869459 PMC1126510

