# Supplementary material for: The association of multiple anti-hypertensive medication classes with Alzheimer’s disease incidence across sex, race, and ethnicity
Source: PLoS One. 2018 Nov 1;13(11):e0206705. doi: 10.1371/journal.pone.0206705 (PMC6211717; doi:10.1371/journal.pone.0206705)
Supplement: S8 Table — Logistic regression results with sample of 2009–2013 Medicare enrollees with use of antihypertensive (AHT) prescription drugs (angiotensin converting enzyme inhibitors (ACEIs), angiotensin-II receptor blockers (ARBs), beta-blockers, calcium channel blockers, loop diuretics, and thiazide diuretics). Each pair of ORs (for ACEI and ARB) represents results from a separate regression, which compares ACEI and ARB users to non-RAS users in the same sex-race/ethnicity subgroup. Users defined as those with 180 possession days and 2 claims in year t-1 and year t-2. Sample restricted to person-years with 3 years fee-for-service, 3 years Part D, age 67+, no deaths in the reference year (year t), no prior AD diagnoses, and no prior use of acetylcholinesterase inhibitors (AChEIs) or memantine. RAS (renin angiotensin system) acting drugs are ACEIs and ARBs. Controls are age, age squared, sex, race, education, income quartiles, statin use (t-1), years since hypertension diagnosis, HCC comorbidity index, number of physician visits, and indicators for past diagnoses of diabetes, atrial fibrillation, acute myocardial infarction, stroke, and hyperlipidemia. Standard errors are clustered at the county level. (DOCX) [file pone.0206705.s008.docx]

| **S8 Table: Odds ratios (with 95% CI) of AD incidence associated with use of ACEI and ARBs, relative to non-RAS users, with users defined at 180 possession days** | | | | | |
| --- | --- | --- | --- | --- | --- |
|  |  |  |  |  |  |
|  |  | All | White | Black | Hispanic |
| Females | ACEI | 1.021, p=0.126 | 1.023, p=0.148 | 1.008, p=0.860 | 0.957, p=0.375 |
|  |  | CI: 0.994-1.049 | CI: 0.992-1.054 | CI: 0.924-1.100 | CI: 0.869-1.054 |
|  | ARB | 0.945, p<0.001 | 0.939, p=0.001 | 0.914, p=0.071 | 1.008, p=0.880 |
|  |  | CI: 0.917-0.975 | CI: 0.906-0.973 | CI: 0.829-1.008 | CI: 0.910-1.116 |
| Males | ACEI | 0.982, p=0.438 | 0.978, p=0.381 | 1.045, p=0.602 | 0.948, p=0.634 |
|  |  | CI: 0.938-1.028 | CI: 0.932-1.027 | CI: 0.887-1.230 | CI: 0.760-1.182 |
|  | ARB | 0.839, p<0.001 | 0.843, p<0.001 | 0.893, p=0.372 | 0.905, p=0.386 |
|  |  | CI: 0.787-0.896 | CI: 0.785-0.904 | CI: 0.698-1.144 | CI: 0.723-1.134 |
| Logistic regression results with sample of 2009-2013 Medicare enrollees with use of antihypertensive (AHT) prescription drugs (angiotensin converting enzyme inhibitors (ACEIs), angiotensin-II receptor blockers (ARBs), beta-blockers, calcium channel blockers, loop diuretics, and thiazide diuretics). Each pair of ORs (for ACEI and ARB) represents results from a separate regression, which compares ACEI and ARB users to non-RAS users in the same sex-race/ethnicity subgroup. Users defined as those with 180 possession days and 2 claims in year t-1 and year t-2. Sample restricted to person-years with 3 years fee-for-service, 3 years Part D, age 67+, no deaths in the reference year (year t), no prior AD diagnoses, and no prior use of acetylcholinesterase inhibitors (AChEIs) or memantine. RAS (renin angiotensin system) acting drugs are ACEIs and ARBs. Controls are age, age squared, sex, race, education, income quartiles, statin use (t-1), years since hypertension diagnosis, HCC comorbidity index, number of physician visits, and indicators for past diagnoses of diabetes, atrial fibrillation, acute myocardial infarction, stroke, and hyperlipidemia. Standard errors are clustered at the county level. | | | | | |
|  |  |  |  |  |  |
|  |  |  |  |  |  |
|  |  |  |  |  |  |
|  |  |  |  |  |  |
|  |  |  |  |  |  |
|  |  |  |  |  |  |
|  |  |  |  |  |  |
|  |  |  |  |  |  |
